# Patient‐Reported Outcomes Following Loss of Permanent Incisors due to Traumatic Dental Injuries: A Retrospective Cross‐Sectional Pilot Cohort Study

**DOI:** 10.1002/cre2.70328

**Published:** 2026-03-31

**Authors:** Josephine Solgaard Henriksen, Eva Lauridsen, Simon Storgård Jensen, Nuno Vibe Hermann

**Affiliations:** ^1^ Pediatric Dentistry and Clinical Genetics, Department of Odontology, Faculty of Health and Medical Sciences University of Copenhagen Copenhagen Denmark; ^2^ Department of Oral and Maxillofacial Surgery Copenhagen University Hospital, Rigshospitalet Copenhagen Denmark; ^3^ Oral Surgery, Research Section for Oral Biology and Immunopathology, Department of Odontology, Faculty of Health and Medical Sciences University of Copenhagen Copenhagen Denmark

**Keywords:** patient‐reported outcomes, quality of life, tooth loss, traumatic dental injuries, treatment

## Abstract

**Purpose:**

This study aimed to investigate the effects of trauma‐related tooth loss before age 18 years on adolescents and young adults.

**Materials and Methods:**

Ninety‐three patients with permanent incisor loss due to traumatic dental injury (TDI) completed a newly developed pilot, TDI‐specific questionnaire evaluating their experiences following the loss of a permanent incisor. Descriptive statistics were used to analyze responses, and multiple linear regression was used to examine the effects of age and sex.

**Results:**

Patient‐reported outcome measures indicated that 74.2% of the respondents reported an impact of tooth loss on their appearance. The mean score for perceived esthetic impact was 3.62 on a 0–10 scale. Despite treatment completion, 78.5% of patients reported recurring thoughts regarding their tooth loss, and 81.7% indicated that, to varying degrees, their tooth loss imposed limitations on their social interactions. Dissatisfaction with temporary tooth replacements was reported by several patients. Among the 93 patients, 58 (62.3%) had received their permanent tooth placement and expressed very high satisfaction with this solution (mean score: 9.05 on a 0–10 scale). Overall, 92.5% of the participants reported satisfaction with the communication during the treatment. Sex significantly affected the patient responses regarding esthetic appearance and satisfaction with the course of treatment.

**Conclusion:**

This patient‐reported outcome‐based study indicates that trauma‐related tooth loss occurring at a young age adversely affects the oral health‐related quality of life of adolescents and young adults, even after treatment completion, thereby impacting their overall quality of life.

## 1 Introduction

Traumatic dental injuries (TDIs) are common, with oral injuries accounting for 5% of all bodily injuries across all ages (Petersson et al. 1997; Andreasen et al. 2018). A meta‐analysis conducted in 2018 found that TDIs would rank as the fifth most common condition if included among the 300 most significant acute and chronic diseases and injuries identified by the 2015 Global Burden of Disease study (Global regional and national incidence prevalence and years lived with disability for 310 diseases and injuries 1990–2015: a systematic analysis for the Global Burden of Disease Study 2015 regional and national incidence prevalence and years lived with disability for 310 diseases and injuries 1990–2015: a systematic analysis for the Global Burden of Disease Study 2015 2016; Petti et al. 2018). The maxillary central incisors are reported to be the most commonly affected teeth (70%) (Andreasen et al. 2018). One study demonstrated that 30% of children had sustained injuries in the primary dentition, whereas 22% had sustained injuries to the permanent dentition (Andreasen and Ravn 1972).

TDIs can be classified into nine types of fractures and six types of luxation entities. However, combination injuries, in which luxation and fractures occur simultaneously, are common. A total of 54 possible combinations were identified. Each of these combinations must be considered as a distinct healing scenario (Andreasen et al. 2012, 2019). Each trauma scenario, whether solitary or combined, is associated with different treatment approaches, prognoses, and risks of complications. Although most TDIs generally exhibit a favorable prognosis, some cases are associated with poor long‐term outcomes. The primary objective of TDI treatment is to ensure the long‐term survival and success of the affected tooth while preventing complications that could compromise subsequent restorations or the supporting bone or soft tissues (Andreasen et al. 2018; Bourguignon et al. 2020).

Several alternatives are available for tooth replacement following tooth loss. These include orthodontic space closure, autotransplantation, resin‐bonded bridges, or tooth‐ or implant‐supported fixed dental prosthesis (FDP) (Andreasen et al. 2019). The choice of treatment depends on the individual patient's situation and the extent of trauma.

Furthermore, the fact that TDI occurs most often in young patients, whose facial skeleton continues to grow, permits the use of certain treatment methods while contraindicating others. As the “best available treatment” is selected for each patient, no single treatment can be considered the first choice for replacing missing anterior teeth. Despite the availability of various treatment options, the goal is to achieve a result that is esthetically pleasing, simple, clinically acceptable, biologically sound, and cost‐effective (Andreasen et al. 2019; Nørgaard Petersen et al. 2022). Irrespective of the treatment modality chosen, replacement of the central incisors following TDI remains a clinical challenge that often extends over several years (Andreasen et al. 2018, 2019; Bourguignon et al. 2020; Nørgaard Petersen et al. 2022; Freire‐Maia et al. 2015). Hence, tooth loss frequently leads to bone resorption, soft tissue recession, and scar tissue formation in the affected area. The highest degree of bone resorption is seen in the labial bone in the anterior maxilla (Chappuis et al. 2017). The management of these complex cases requires interdisciplinary collaboration within a highly specialized treatment team.

The World Health Organization (2025) defines quality of life as “individuals' perceptions of their position in life in the context of the culture and value systems in which they live and in relation to their goals, expectations, standards and concerns.” Oral health‐related quality of life (OHRQoL) describes the extent to which oral health or disease influences an individual's daily life, social interactions, self‐esteem, and perceptions of their oral health, thereby affecting their overall quality of life (Das et al. 2022; Tewari et al. 2023; World Health Organization 1998; Sischo and Broder 2011).

Traditionally, treatment outcomes in dental traumatology have been assessed based on clinical outcomes. However, recent emphasis has shifted toward assessing outcomes from the patient's perspective (Freire‐Maia et al. 2015; Das et al. 2022; Tewari et al. 2023; Nagendrababu et al. 2023; John 2018; Borges et al. 2017; Antunes et al. 2020; Lopez et al. 2019; Zaror et al. 2018; Kenny et al. 2018). The term “patient‐related outcome” (PRO) refers to any self‐reported description of a patient's health status without interpretation by clinicians or other third parties (Nagendrababu et al. 2023). Patient‐reported outcome measures (PROMs) are the instruments to assess PRO's. The idea is to gain insights into the patient‐perceived disease impact, enabling clinicians to better understand patients' experiences, priorities, preferences, values, and expectations (Tewari et al. 2023; Nagendrababu et al. 2023; John 2018). Nonetheless, a recent narrative review by Nagendrababu et al. (2023), summarizing the current use of PROs and PROMs within dental traumatology, concluded that the development of a novel, field‐specific assessment tool is required to more accurately capture the perspectives of patients undergoing treatment following TDIs.

Various types of PROs and PROMs have been employed to assess the psychological impact of TDIs and their effect on OHRQoL. However, the quality of life remains challenging due to its inherently subjective nature (Freire‐Maia et al. 2015; Das et al. 2022).

A systematic review published in 2022 concluded that TDIs affecting permanent teeth strongly influence OHRQoL in children and adolescents (Das et al. 2022). Furthermore, the review demonstrated that the treatment of TDIs considerably improved OHRQoL. Specifically, treatment not only improved functional abilities, such as eating, biting, and speaking, but also social and emotional well‐being as well as activities of daily living, including smiling, displaying teeth without embarrassment, and engaging comfortably in social interactions (Das et al. 2022).

TDIs may affect individuals of both sexes and all age groups; however, women appear to experience a greater negative impact compared with men (Das et al. 2022). With regard to age, most studies have focused on “children,” “pre‐schoolers,” “school children,” and “adolescents” under 19 years of age (Borges et al. 2017; Antunes et al. 2020; Lopez et al. 2019; Zaror et al. 2019). To the best of our knowledge, no association has been established between patient age and OHRQoL. Hence, expanding the age range to include individuals aged > 19 years could provide valuable insight into whether age significantly influences PROs.

An umbrella review published in 2023, which aimed to evaluate the impact of TDIs on OHRQoL, identified the lack of primary studies. The majority of existing studies were conducted in a single country (Brazil [82%]), with considerable overlap and heterogeneity among the studies (Tewari et al. 2023). Nonetheless, TDIs appear to exert a significant impact on OHRQoL in children and adolescents when different types of injuries are collectively considered (Tewari et al. 2023). A general need exists for further studies investigating the effects of TDIs both immediately following trauma and in the years after completion of treatment.

## 2 Aim of the Study

Thus, this study aimed to evaluate the occurrence of trauma‐related tooth loss in individuals aged < 18 years and to assess how the course of treatment influences PROMs using a newly developed pilot, TDI‐specific questionnaire. We hypothesized that trauma‐related tooth loss significantly affects patients. Furthermore, it was assumed that age and sex have a significant impact on patients' perceptions of limitations in social situations, esthetic appearance, and satisfaction with treatment course and permanent tooth replacement.

In addition, this study should be regarded as an initial step toward the development of a TDI‐specific PROM, providing pilot data to inform future questionnaire validation studies.

## 3 Materials

The study was conducted in accordance with the guidelines of the Declaration of Helsinki 2013. Approval to conduct the study, as well as permission to store and handle the data, was granted by the regional scientific ethics committee^1^ and the University Hospital.^2^ All data were anonymized and collected in 2024.

The study material included children and adolescents who sustained TDIs involving the upper anterior dentition, resulting in the loss of one or more permanent incisors. All patients were initially treated and followed up at the Municipal Pediatric Dental Care Centre in the capital region of Denmark. Referral to the Regional Dental Care Department of Oral and Maxillofacial Surgery, University Hospital Rigshospitalet, Copenhagen, Denmark, was made due to tooth loss caused by TDI.

Furthermore, patients who fulfilled the following criteria were included in the study:

1. Loss of at least one permanent incisor before the age of 18 years as a result of dental trauma occurring between 2011 and 2021.
2. All TDI diagnoses resulting in tooth loss.
3. Availability of tooth‐specific clinical information and radiographs from the time of injury and subsequent follow‐up visits conducted according to a standardized protocol.
4. Informed consent was obtained from all patients. For minors, consent was provided by their custodial parents or legal guardians, who also received written information regarding the study.

Some patients received permanent tooth replacement, whereas others still had a temporary tooth replacement solution. In both groups, the tooth replacement was performed by the Regional Dental Care Department.

### 3.1 Patient‐Reported Outcome (PRO)

To assess patient‐reported outcomes (PRO), a new questionnaire was developed. The development process followed established methodological steps (Rattray and Jones 2007; Tsang et al. 2017; Jokovic et al. 2002):

- **Preliminary considerations:** Existing validated instruments, such as the Oral Health Impact Profile (OHIP) and Child Perceptions Questionnaire (CPQ), were reviewed. However, none were deemed suitable for capturing the impact of trauma‐related tooth loss before age 18 years on adolescents and young adults.
- **Development of a TDI‐specific questionnaire:**
  ◦ Internal expert group formation: Comprised of the authors of this article.
  ◦ Determination of the format: To minimize response bias, self‐administration was chosen. Moreover, to avoid dropouts due to transportation or lack of time, the questionnaire should be able to be completed quickly and easily electronically.
  ◦ Item format selection: Primarily close‐ended items (e.g., true/false, Likert scales, rating scales) were used to facilitate ease of administration and analysis. However, open‐ended items were included to allow patients to provide additional qualitative input.
  ◦ Item development: The questionnaire items were formulated in Danish and designed to be easily understandable for adolescents and young adults across a broad age range.
  ◦ Initial clinical review: An internal clinical review was conducted by the author group, all of whom have clinical and research experience within dental traumatology. The review aimed to ensure clinical relevance and appropriate coverage of patient‐reported domains related to trauma‐related tooth loss. This step was intended to refine item wording and scope rather than to establish formal content validity. The process resulted in a 13‐item digital questionnaire consisting of 12 close‐ended items and one open‐ended item.
  ◦ Preliminary pilot testing (feasibility testing and face validity assessment): Ten patients with trauma‐related tooth loss completed the questionnaire as part of preliminary pilot testing. This phase served to assess feasibility and face validity by identifying unclear wording, ambiguous formulations, and potential barriers to completion. The patients provided structured feedback regarding clarity, comprehensibility, and acceptability of the items. Based on this feedback, minor linguistic adjustments were made to improve item wording and ensure that the questions were easily understood by patients across different age groups. No items were added or removed during this phase.
  ◦ Feasibility testing in the study population: The final version of the questionnaire (Table 1) was distributed to the study population as part of the present feasibility study (Tsang et al. 2017; Jokovic et al. 2002). Participants under 18 were carefully instructed to complete the questionnaire in consultation with their custodial parents or legal guardians. All participants were informed that the study coordinator (JSH) could be contacted at any time for further information regarding the study or clarification of the questionnaire.

**Table 1:** Responses to the questionnaire and corresponding scores.

Questionnaire questions	Results
1. How much pain and discomfort have affected you in the first weeks after your dental injury on a scale from 0 to 10? (0 = no pain and 10 = worst possible pain) If you cannot remember, check this box [].	Mean score: 6.40
	Standard deviation: 3.25
	Note: A total of 18 of 93 participants responded that they could not recall the pain and discomfort level as the injury occurred several years ago.
2. How much pain or discomfort do you currently experience due to dental injury? (0 = no pain and 10 = worst possible pain).	Mean score: 1.14
	Standard deviation: 2.07
	In total, 61.3% of the participants reported no current discomfort or pain.
	Approximately 38.7% of the participants reported some level of discomfort. This group was a mix of patients who had received their permanent tooth replacement solution and patients who were still waiting for their permanent tooth replacement.
3. On a scale from 0 to 10, how much has your tooth loss limited you in social situations? (0 = has not limited me at all; 10 = has limited me a lot)	Mean score: 4.01
	Standard deviation: 3.00
	Approximately 81.7% of the participants experienced some degree of social limitation related to their tooth loss.
4. How satisfied were you with your temporary tooth replacement on a scale of 0–10? (0 = not satisfied at all; 10 = very satisfied) (i.e., the temporary dental replacement—such as a denture or dental crown—that you had or have for a period before the final treatment was performed or needs to be performed)	Mean score: 5.85
	Standard deviation: 2.91
5. Have you changed or needed to change your diet because of your temporary tooth replacement? (Yes or No)	Yes: 46 (49.5%)
	No: 47 (50.5%)
6. On a scale from 0 to 10, how much have dental visits related to your tooth injury interfered with your work (did you miss out on work or school)? (0 = no interference; 10 = has interfered/missed a lot of work/school due to dental visits)	Mean score: 4.59
	Standard deviation: 3.08
7. How frequently do you think about having lost a tooth? (1 = never, 2 = sometimes, 3 = often, and 4 = every day or almost every day)	Mean score: 2.55
	Standard deviation: 1.12
	Distribution of scores of all 93 responders:
	1: 20 (21.5%)
	2: 29 (31.2%)
	3: 17 (18.3%)
	4: 27 (29%)
	Distribution of scores of the 58 responders who have received the permanent tooth replacement:
	1: 18 (31%)
	2: 19 (32%)
	3: 11 (19%)
	4: 10 (17.2%)
8. Do you feel that others notice a difference in the appearance of your teeth? This applies regardless of whether you have your temporary or permanent tooth replacement (Yes or No)	Yes: 22 (23.7%)
	No: 71 (76.3%)
9. On a scale from 0 to 10, how much do you feel your appearance has been affected by the dental injury? (0 = no effect on my appearance, 10 = greatly affected my appearance)	Mean score: 3.62
	Standard deviation: 3.18
10. How satisfied are you with the course of treatment on a scale of 0–10? (0 = not satisfied at all; 10 = very satisfied)	Mean score: 7.46
	Standard deviation: 2.34
	Distribution of scores:
	– Approximately 7.7% of the participants responded with a score between 0 and 3. – Approximately 16.2% of the participants responded with a score between 4 and 6. – Approximately 76.4% of the participants responded with a score between 7 and 10.
11. During the treatment, did the dentist explain your tooth condition and the long‐term treatment plan enough for you to understand? (Yes or No) (e.g., explanations about why the tooth needed extraction or later fell out/broke, or why you could not get the final dental replacement immediately but needed to use a temporary one first)	Yes: 86 (92.5%)
	No: 7 (7.5%)
12. How satisfied are you with your permanent tooth replacement on a scale of 0–10? (0 = not satisfied at all; 10 = very satisfied)	Mean score: 9.05*
	*Based on the responses from 58 participants, 35 had not yet received their final tooth replacement.
– If you have not yet received your final tooth replacement, please skip this question.	
	Distribution of the 58 permanent tooth replacement solutions:
	– 55 implants – 2 tooth‐supported bridges – 1 autotransplantation
13. What do you think dentists can improve in the future when treating patients who have experienced dental trauma? (Open‐ended response)	

*Note:* English translation of the 13 questions.

A total of 235 patients who met the inclusion criteria were invited to participate by completing a questionnaire consisting of 13 questions.

### 3.2 Statistical Methods

Descriptive statistics were used to analyze the questionnaire responses. Furthermore, multiple linear regression was used to analyze questions 3, 9, 10, and 12 to assess whether patient sex or age significantly influenced on the responses. All statistical analyses were performed using the SPSS software (version 29.0.1.0). A *p*‐value of 0.05 was considered significant.

## 4 Results

A total of 93 out of 235 patients responded to the questionnaire, corresponding to a response rate of 39.6% (men: 48; women: 45). Participants were aged between 14 and 31 years, with a mean age of 24.77 years (standard deviation [SD] = 5.242). Among non‐responders (*N* = 142), the mean age was 24.09 years (SD = 4.86), and 31.7% were female.

The translated patient questionnaire and results are presented in Table 1. The results from questions 3, 9, 10, 12, and 13 are elaborated below. Table 2 presents the results of the statistical analysis of questions 3, 9, 10, and 12.

**Table 2:** Multiple linear regression analysis of questions 3, 9, 10, and 12 to assess whether patient gender or age significantly influenced the responses.

		Estimate	*p* value	95% confidence interval
Question 3	Gender	0.183	0.771	−1.064 to 1.430
	Age	0.052	0.390	−0.068 to 0.710
Question 9	Gender	1.815	0.006	0.543–3.086
	Age	−0.041	0.503	−0.163 to 0.081
Question 10	Gender	−1.075	0.027	−2.025 to 0.125
	Age	0.031	0.496	−0.060 to 0.122
Question 12	Gender	−0.378	0.456	−1.389 to 0.631
	Age	0.10	0.117	−0.026 to 0.227

*Note:* Link function: Logit. In the gender analysis, women are compared with men. The results are shown in log odds.

Question 3:

Age (*p*‐value = 0.390) and sex (*p*‐value = 0.771) did not affect social situations.

Question 9:

Sex was found to significantly influence responses (*p*‐value = 0.006). Female respondents had an average score that was 6.1 times higher than the average score of male respondents.

Question 10:

Only sex had a significant effect on patient responses (*p*‐value = 0.027). Female respondents had an average score that was 2.9 times lower than that of male respondents.

Question 12:

Age and sex did not have a significant effect on the responses.

The mean duration since the placement of the permanent tooth solution was calculated to be 4.36 years, corresponding approximately to the year 2019 (SD: 2.9 years). No significant correlation was found between the number of years since treatment and patient satisfaction with the permanent tooth replacement solution (*p*‐value = 0.34).

Question 13:

Question 13 consisted of a free‐text response, precluding the generation of descriptive statistics.

Examples of advice provided to the patients are as follows:

“Take the time to align expectations with the patient at the very beginning of the process.” “Remember that tooth loss is both a significant physical and psychological trauma; show empathy toward the patient.” “Provide continuous updates on treatment progress and the expected timeline for initiation.” “The waiting list for permanent tooth replacement is excessively long.” “Refer patients sooner to Regional Dental Care. Dentists in the municipality appear to lack sufficient training in managing TDIs. Only after being referred to Regional Dental Care did I feel safe and confident that my treatment was properly managed.”

## 5 Discussion

The primary finding of this study was that trauma‐related tooth loss occurring at a young age significantly impacts an individual's OHRQoL and, consequently, their overall quality of life.

Most patients reported that their tooth loss had restricted their social interactions. This effect was especially pronounced among women, who more frequently perceived a negative impact on their esthetic appearance.

Several patients expressed dissatisfaction with temporary tooth replacement options, particularly removable prostheses such as dentures. However, the overall satisfaction with the course of treatment, communication during treatment, and the permanent tooth replacement solution remained high.

For questions 3 and 12, the data fail to reject the hypothesis that there is no effect of age and gender. In questions 9 and 10, we found that the data fail to reject the hypothesis that there is no effect of age, and the data reject the hypothesis that there is no effect of gender.

The following sections discuss how patients are affected and to what extent.

### 5.1 Pain Level

Pain perception is inherently subjective, and this was expected to be reflected in the analysis. Additionally, as the injury occurred several years prior, a certain degree of recall bias in the results was expected.

However, the purpose of assessing pain levels was to gain insights into how patients remember their injury (i.e., the patient's recollection of the traumatic event). The patients had varying diagnoses, which may account for the differences in reported pain and discomfort. However, the pain intensity does not always correlate with the severity of injury. Additionally, it can be influenced by individual experiences and psychological factors (Khan et al. 2019; Raja et al. 2020). Nonetheless, TDI appeared to cause a relatively high level of pain and discomfort during the initial weeks following the injury. As expected, the mean pain score for question 2 was lower than that for question 1, a finding that was confirmed by the results.

### 5.2 Time Consumption

The number of dental visits varies depending on the diagnosis and complexities of individual cases and treatments. This resulted in considerable individual variability, as reflected by the relatively high standard deviation. A retrospective study which investigated the factors that influence the number of visits per tooth following TDI to the permanent dentition reported a median of six visits per tooth, with a range of 1–22 visits. This study included 186 teeth from 100 patients (Keasberry et al. 2013).

One possible explanation for the mean score is that, in many cases (58 out of 93), patients had already received their permanent tooth replacement solution. Consequently, these patients likely only require annual checkups, which are not particularly time‐consuming. Given that the mean age was 24.77 years, some patients probably experienced financial impacts due to absence from work. This patient group was expected to score higher on the scale, but the data did not confirm this. Repeated absences from school or education, even without financial consequences, may have contributed to a higher score on Question 6.

In Denmark, dental care is free for children and adolescents up to 21 years of age. However, checkups at regional dental care remain free regardless of age. Patients likely appreciate this service as a valuable opportunity to receive highly qualified care without cost, which may contribute to their perception of it as convenient rather than time‐consuming.

### 5.3 Awareness and Concerns

Most patients (78.5%) reported thinking about their tooth loss to some degree. Among the 27 patients who thought about their tooth loss daily or almost daily, one might expect a direct correlation to whether the patients had received their permanent tooth replacement or not. However, 10 of these 27 patients had already received their permanent replacement. Therefore, awareness of previous tooth loss can persist even after treatment. It is seen that there is a tendency to score lower after completing treatment. This finding underscores the importance of addressing such ongoing awareness when managing patients with a history of TDI. These results align with those of a previous retrospective, cross‐sectional cohort study (Henriksen et al. 2025).

Moreover, 81.7% of the participants reported experiencing limitations in social activities due to their tooth loss. The extent of these social limitations varied widely, with 24.8% of the participants rating their reluctance to engage in social events between 7 and 10, indicating a high level of embarrassment related to their partial edentulism.

Approximately one‐quarter of the patients reported that others had noticed a difference in the appearance of their traumatized teeth. Unfortunately, these responses could not be distinguished based on whether the patient had received their permanent tooth replacement or not. By contrast, another quarter of the participants reported feeling unaffected by their appearance. This suggests that many participants perceived a greater impact of their dental trauma on themselves than was recognized by those around them.

Fifty‐eight of the 93 patients had received their permanent tooth replacement and expressed satisfaction with this solution. The relatively low mean score for Question 9 may be explained by the potential correlation between getting one's permanent tooth replacement and the degree to which patients feel cosmetically affected. Question 9 might have been more informative if divided into two parts: one assessing the cosmetic impact before permanent tooth replacement, and another assessing the impact after treatment.

### 5.4 Treatment and Patient Satisfaction

**Figure 1:**
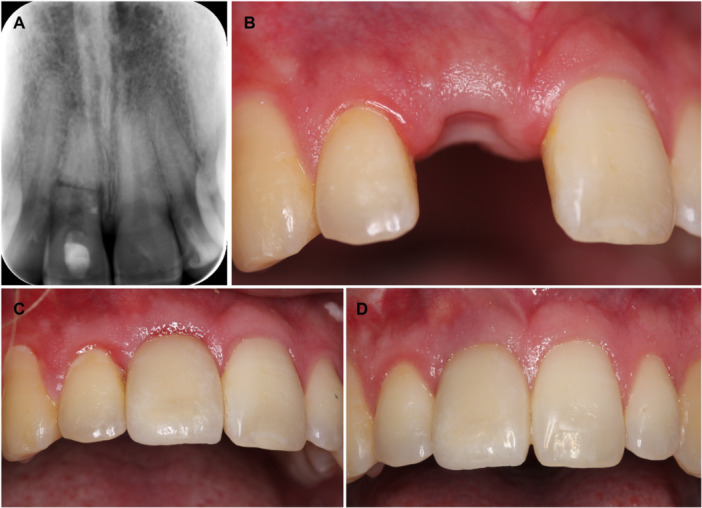
(A–D) Patient case with trauma‐related tooth loss of 11 after a root fracture (location of fracture; cervical/mid‐root) that happen when the patient was 17 years old. Due to pulp necrosis, the coronal fragment has a root canal treatment. After repeated infection in the area despite sufficient endodontic treatment, the tooth was extracted. A denture served as the temporary tooth replacement. After completion of facial skeletal growth, the patient received an implant as a permanent tooth solution at the regional dental care. (A) Radiograph taken 7 years after the TDI. There are clear signs of infection in the coronal fragment of 11. At this point, the old endodontic filling was removed, and calcium hydroxide was placed as an intracanal medicament. A new root canal treatment was performed. (B) Clinical photo after tooth extraction of 11. The patient is 25 years old at this point. (C) Clinical photograph after placement of the dental implant in region 11. (D) Clinical photo at the 5‐year follow‐up control at the regional dental care. The patient is very satisfied with the esthetic appearance of 11.

Overall satisfaction with the course of treatment was relatively high. A significant effect of sex on patient responses was observed, with women reporting lower satisfaction compared with men. This finding aligns with the greater cosmetic impact experienced by women. Figure 1 shows a patient case where the patient was satisfied with the esthetic outcome and course of treatment.

Clinicians should be aware of these gender differences. To enhance patient satisfaction, clinicians should prioritize expectation alignment to avoid misunderstandings and ensure a shared understanding of what expectations are realistic and achievable. Through an empathic and transparent communication, individualized treatment plans can be made. In addition, ongoing evaluation and adjustment of expectations might be beneficial for cooperation and long‐term satisfaction.

A high proportion of the patients felt that the treatment plan was explained to them in an understandable manner throughout the process. Achieving a high level of patient satisfaction naturally depends on clear and effective communication between healthcare professionals and patients. No common features were identified among the seven patients who reported dissatisfaction with communication, making it difficult to determine the specific reasons or points where communication may have failed.

### 5.5 Temporary Tooth Replacement

**Figure 2:**
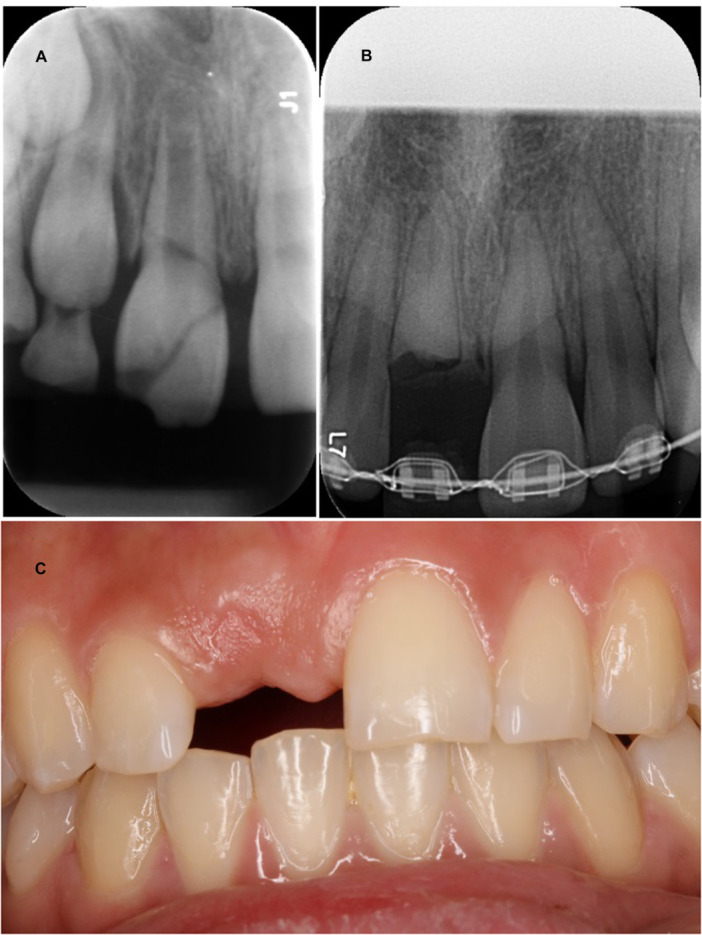
(A–C) Patient case: 11 were diagnosed with crown‐root fracture with pulpal involvement when the patient was 8 years old. (A) Radiograph from the day of the injury before initial treatment. Due to the severity of the fracture, the tooth could not be restored. The patient was referred to the Regional Dental Care, Department of Oral and Maxillofacial Surgery, Copenhagen University Hospital. To prevent resorption of the labial bone in the anterior maxilla, decoronation of 11 was performed. Following consultation with the interdisciplinary team, the treatment plan identified a dental implant as the most appropriate permanent solution. This required more space to be created in region 11. Additional space was created through orthodontic treatment. (B) Radiograph taken at the 8‐year follow‐up. 11 is seen decoronated, and the patient is undergoing orthodontic treatment with fixed equipment. (C) Clinical photo taken at the 12‐year follow‐up performed at the regional dental care. The patient is now 20 years old and has a denture. Due to continuous facial skeleton growth, the patient is still waiting for the permanent tooth replacement—in this case, an implant.

A significant proportion of participants expressed dissatisfaction with their temporary tooth replacements. The most commonly used temporary solution in this study was dentures. Alternative approaches included fixation of the traumatized tooth crown to the adjacent teeth, resin‐bonded bridges, or intermediate teeth supported by palatal temporary anchorage devices. Some patients had undergone several different temporary solutions over the years. Removable options, in particular, were frequently associated with high levels of dissatisfaction. Many patients were still undergoing skeletal or craniofacial growth, which limits the feasibility of certain temporary solutions. Although patients may understand this limitation, accepting it can be challenging. Figure 2 illustrates one patient currently using a denture who nonetheless reported relatively high satisfaction with the temporary tooth replacement. This assessment was based on their responses to question 4, where the patient provided a score of 8 on a scale from 0 to 10.

More than 50% were forced to change their dietary habits because of the temporary tooth replacement.

### 5.6 Permanent Tooth Replacement

In general, patients report high satisfaction with the esthetic outcomes of autotransplanted teeth in the anterior maxilla, resin‐bonded bridges, implant‐supported single crowns, and orthodontic space closure (Bawa et al. 2023; Vilhjálmsson et al. 2011; Jamilian et al. 2015; Thilander 2008; Akhlef et al. 2018). The present study supports these findings: 33 out of 58 who have reviewed their permanent tooth replacement reported being “very satisfied,” corresponding to a score of 10 on the scale. Neither age nor sex significantly influenced their responses.

**Figure 3:**
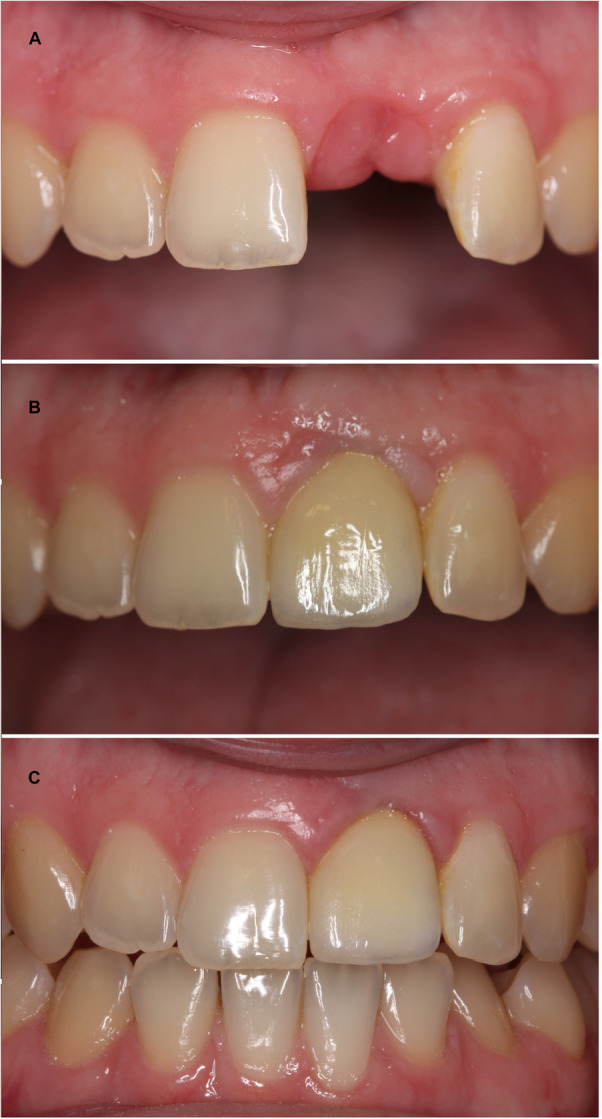
(A–C) Patient case with trauma‐related tooth loss of 21. After a severe TDI then the patient was 8 years old, 21 became ankylosed and infra‐positioned. The tooth was decoronated to preserve the alveolar bone in the area. A denture served as the temporary tooth replacement. After completion of facial skeletal growth, the patient received an implant as the permanent tooth solution. (A) Image shows a clinical photo before tooth replacement of 21. (B) Image shows a clinical photo at 1‐year follow‐up. The patient is satisfied with the esthetic appearance at this point, and (C) shows the clinical photo at 5‐year follow‐up control. Due to the continued eruption of adjacent teeth, even after skeletal growth has ceased, the implant is now seen infra‐positioned (1–2 mm). The patient is not satisfied with the esthetic appearance now.

Considering this, high patient satisfaction appears reasonable. Several studies have reported high patient satisfaction with implant‐supported single crowns (Nørgaard Petersen et al. 2022; Gotfredsen 2012; Andersson et al. 2003). A systematic review of implant treatment following traumatic tooth loss also found high survival rates for implants and their superstructures, along with consistently positive patient‐reported outcomes (Nørgaard Petersen et al. 2022). These findings align with the overall survival rates observed in dental implant treatments in general (Jung et al. 2012; Terheyden and Wüsthoff 2015). However, a systematic review reported a gap in the literature regarding long‐term esthetic outcomes of dental implants after TDI (Nørgaard Petersen et al. 2022). In this context, distinguishing between implant “survival” and “success” remains essential. For example, one well‐known challenge of dental implant treatment in the anterior maxilla is the continued eruption of adjacent teeth, even after skeletal growth has ceased, leading to implant infraposition (Nørgaard Petersen et al. 2022; Jensen 2019). Such changes undoubtedly impair esthetic outcomes. Future studies addressing this issue are warranted. Figure 3 shows a patient case where continued eruption of the adjacent teeth has led to infraposition of the implant.

A retrospective clinical study evaluating the white and pink esthetics of anterior maxillary implants reported high mean scores in both categories. However, overall patient satisfaction exceeded the objective esthetic ratings assigned by clinicians (Belser et al. 2009).

A 2016 systematic review comparing the biological, functional, and esthetic outcomes of orthodontic space closure and prosthetic replacement options (resin‐bonded bridges, implant‐supported single crowns, and tooth‐supported FDPs) for patients with agenesis of the lateral maxillary incisors reported that orthodontic space closure should be the treatment of choice when all options are feasible (Kiliaridis et al. 2016). Autotransplantation was not included in the analysis. However, it also provides a biological replacement option for young patients with favorable functional and esthetic outcomes (Akhlef et al. 2018). Both approaches appear advantageous for young individuals with ongoing skeletal growth, allowing for earlier treatment completion and a lower prevalence of subsequent biological and technical prosthodontic complications (Nørgaard Petersen et al. 2022; Akhlef et al. 2018; Kiliaridis et al. 2016). Nonetheless, not all patients qualify for orthodontic closure or autotransplantation; therefore, clinicians must individualize treatment choices.

Many patients in the present study received pre‐prosthetic orthodontic treatment to upright and parallel adjacent teeth and/or to gain sufficient space in the mesio‐distal dimension.

One study evaluated the cost‐effectiveness of various tooth replacement options for agenesis of the maxillary lateral incisors (Akhlef et al. 2018). To our knowledge, no similar studies have been conducted on tooth replacement following TDIs. However, one study estimated the annual costs associated with TDIs. This study found that the costs ranged from USD 2 million to USD 5 million per 1 million inhabitants, depending on the treatment scenario (Borum and Andreasen 2001).

One limitation of this study is the use of a non‐validated questionnaire. Although validated questionnaires, such as the Danish version of the Child Perceptions Questionnaire (CPQ11‐14 [Wogelius et al. 2009]) and the Oral Health Impact Profile (OHIP‐14 or OHIP‐49) (Gera et al. 2020; Slade and Spencer 1994), were available, they lack focus on the aspects relevant to this study. Therefore, a dedicated questionnaire was developed to evaluate pain, appearance, social concerns, treatment course, and satisfaction with temporary and permanent tooth replacement after TDI. This approach was supported by a narrative review published in 2023 (Nagendrababu et al. 2023). The present study should be regarded as an initial exploratory step, providing pilot data to inform future development and formal psychometric validation of a TDI‐specific PROM, including assessment of content, construct, and criterion validity.

This study did not adjust for potential confounding factors such as the specific type and complexity of the initial trauma or treatment timing, which may influence the interpretation of the results.

All the patients were referred to the Regional Dental Care Department from the Municipal Pediatric Dental Care. The referral time varied among patients. In some patients, tooth loss occurred immediately or shortly after the TDI, whereas in other patients, tooth loss occurred years later due to treatment failure or healing complications. Nevertheless, this applies to all patients who received at least part of their initial treatment and follow‐up at the Municipal Pediatric Dental Care. Several questionnaire items addressed the overall perceptions and satisfaction with treatment. The primary goal remains to achieve the highest possible level of patient satisfaction.

All municipalities are expected to possess the necessary skills and training to manage all types of TDIs. Nonetheless, regional variations in expertise and quality of care may exist. Patient groups may differ between municipalities.

Another limitation of this study is that not all patients with trauma‐related tooth loss were referred to regional dental care. For example, patients undergoing orthodontic space closure typically complete their treatment within Municipal Pediatric Dental Care and are not referred.

Finally, the retrospective nature and relatively small sample size (*N* = 93) limit the study's generalizability. Furthermore, the potential for non‐response bias should be considered. Furthermore, the potential for non‐response bias should be considered. Although age distributions were similar between responders and non‐responders, a higher proportion of men among non‐responders may have influenced participation patterns. Possible reasons for non‐response include a long time since injury or treatment completion, potentially resulting in limited recall or reduced perceived relevance of participation, the time and effort required to complete an online questionnaire, and additional consent requirements for younger patients. In addition, patients who perceived a limited impact of their condition may have been less inclined to participate. Detailed clinical characteristics were unavailable for non‐responders due to lack of informed consent.

Further research with a larger sample size and multi‐center collaboration is needed before generalizing these findings. Moreover, future studies might utilize prospective longitudinal approaches to monitor changes in PROMs throughout the treatment process (before, during, and after treatment completion).

## 6 Conclusion

This PRO‐based pilot study indicates that early trauma‐related tooth loss (before the age of 18 years) negatively impacts adolescents' and young adults' OHRQoL and, consequently, their overall quality of life.

Despite receiving complete treatment, many patients, especially women, remain preoccupied with their tooth loss and feel that it compromises their esthetic appearance. Additionally, a significant number of patients reported social limitations linked to their tooth loss. Although most patients expressed satisfaction with the treatment course and permanent tooth replacement, dissatisfaction was common regarding temporary tooth replacement solutions.

## Author Contributions

Josephine Solgaard Henriksen, Eva Lauridsen, Simon Storgård Jensen, and Nuno Vibe Hermann conceived the idea. Josephine Solgaard Henriksen led data collection. Josephine Solgaard Henriksen performed the statistical analysis and analyzed the data and in collaboration with/under the guidance of Eva Lauridsen, Simon Storgård Jensen, and Nuno Vibe Hermann. All authors contributed significantly to the writing of the manuscript and have approved this manuscript.

## Funding

The authors received no specific funding for this work.

## Conflicts of Interest

The authors declare no conflicts of interest.

## Data Availability

The data that supports the findings of the study are available from the corresponding author upon reasonable request.

## Endnotes

1 Ethical research committee approval number for the project; H‐22024452.

2 University Hospital approval number: p‐2023‐14807.
